# Pyrrolizidine-Derived Alkaloids: Highly Toxic Components in the Seeds of *Crotalaria cleomifolia* Used in Popular Beverages in Madagascar

**DOI:** 10.3390/molecules26113464

**Published:** 2021-06-07

**Authors:** Anjaramampionona Henintsoa Duvale Solofomalala, Clara Fredeline Rajemiarimoelisoa, Randriamampianina Lovarintsoa Judicael, Hanitra Ranjana Randrianarivo, Danielle Aurore Doll Rakoto, Victor Louis Jeannoda, Ahcène Boumendjel

**Affiliations:** 1 Fundamental and Applied Biochemistry Department, Faculty of Sciences, University of Antananarivo, P.O. Box 906, Antananarivo 101, Madagascar; solofomalalahenintsoa@ymail.com (A.H.D.S.); bouba_lova@yahoo.fr (R.L.J.); ranjanamaso@yahoo.fr (H.R.R.); dad.rakoto@yahoo.fr (D.A.D.R.); victor_jeannoda@yahoo.fr (V.L.J.); 2 Pharmacy Department, Faculty of Medicine, University of Antananarivo, P.O. Box 375, Antananarivo 101, Madagascar; fredeline_rajemi@yahoo.fr; 3 Univ. Grenoble Alpes, INSERM, LRB, 38000 Grenoble, France; 4 Univ. Grenoble Alpes, CNRS, DPM, 38000 Grenoble, France

**Keywords:** *Crotalaria*, Fabaceae, traditional use, pyrrolizidine, alkaloids, usaramine, toxicity

## Abstract

Seeds of *Crotalaria cleomifolia* (Fabaceae) are consumed in Madagascar in preparation of popular beverages. The investigation of extracts from the seeds of this species revealed the presence of high amounts of alkaloids from which two pyrrolizidine-derived alkaloids were isolated. One of them was fully characterized by spectroscopic and spectrometric methods, which was found to be usaramine. Owing to the high toxicity of these alkaloids, issuing a strong warning among populations consuming the seeds of *Crotalaria cleomifolia* must be considered.

## 1. Introduction

*Crotalaria* is a genus of flowering plants that belong to the Fabaceae family that is composed of about 600 species distributed across all continents, among which 400 are distributed in Africa [1]. *Crotalaria* is also known under the name rattlebox. This name takes its origin from the rattle sound made by seeds when the dry pods are shaken. The genus *Crotalaria* is used in traditional medicine as deworming, in malaria treatment, and in management of fever [2,3]. In agriculture, the crotalaria species are used as a pre-plant cover for soybean crops to avoid the spread of parasites [4]. However, *Crotalaria* genus is known for its toxicity. In pastoral regions, several species are known to induce fatal toxicity among grazing cows, horses, and sheep, mainly due to the presence of toxic alkaloids [5,6]. Indeed, according to several reports, the seeds of certain *Crotalaria* genus may contain up to 5% of toxic alkaloids, mostly pyrrolizidine-derived alkaloids known for their severe hepatotoxicity and renal failure, following bio-activation by cytochrome P450 (CYP) enzymes in the liver [7,8].

**Figure 1:**
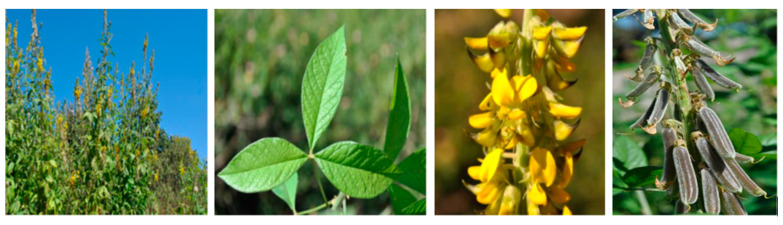
*Crotalaria cleomifolia*. From left to right—aerial part, leaves, flowers, and pods.

In Madagascar, *Crotalaria cleomifolia* is an introduced species from the African continent. It is known by two other names—*Crotalaria longibracteata* and *Crotalaria shumaniana* [9]. *Crotalaria cleomifolia* is a shrub with simple or composed leaves, with yellow or white flowers and fruits in pods (Figure 1) [10]. A field survey revealed that in the Manjakandriana region, near the capital Antananarivo, the leaves and the stems of *Crotalaria cleomifolia* are used as green fertilizers, whereas the crushed seeds are used in the preparation of a coffee substitute [11]. To date, the seeds of *C. cleomifolia* have never been investigated for their phytochemical composition. In this context, we were interested in the seeds that are used in the traditional beverage preparations. Based on the well-established literature, we focused our attention on the potential presence of toxic pyrrolizidine-derived alkaloids that may seriously compromise the traditional use of the seeds of *C. cleomifolia*. Herewith, we report the extraction, purification, and structural identification of alkaloids from the seeds of *C. cleomifolia*.

## 2. Results

### 2.1. Extraction and Purification of Alkaloids

Two parallel extractions (starting from 50 g of grounded seeds) were realized, following classic procedures that rely on the basic character of alkaloids (see Section 4) [12]. The extraction in acidic conditions provided almost a twofold higher quantity of crude alkaloids as compared to the extraction in basic conditions (136 mg versus 66 mg, respectively), with roughly the same chemical composition according to the TLC and HPLC analyses. The purification of the crude extracts was performed on a silica gel chromatography column, using a gradient of dichloromethane:methanol to provide two pure fractions (44 mg and 39 mg), each containing one pure compound, namely, compounds **1** and **2**.

### 2.2. Structural Identification

**Figure 2:**
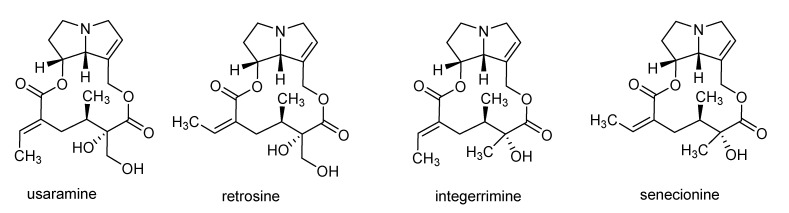
Structure of compound **1** (usaramine), retrosine, integerrimine, and senecionine.

Compound **1** (44 mg) was analyzed by IR, mass spectrometry, and NMR (1D and 2D). The IR spectrum showed the presence of hydroxyl (3466 cm^−1^) and carbonyl (1716 cm^−1^) groups (see experimental section and Supplementary Materials for the IR spectrum). The compound was assigned the molecular weight and molecular formula of 351 g/mol and C_18_H_25_NO_6_, respectively, as determined by HRMS (see Supplementary Materials). The ^13^C NMR showed 2 carbonyls (176.1 and 170.5 ppm), 4 olefinic carbons (133.6–136.6 ppm), 4 saturated carbons linked to oxygen (68.2–82.5 ppm), and two methyl groups (12.4 and 14.3 ppm) (^13^C NMR data are provided in the experimental section and in Supplementary Materials). The ^1^H NMR data were in agreement with the information obtained from the ^13^C NMR spectrum and showed a profile similar to those of pyrrolizidine-alkaloids (^1^H NMR data are provided in the experimental section and in Supplementary Materials). Homo and hetero-2D NMR brought more evidence and confirmed that the compound was in agreement with the known macrocyclic pyrrolizidine alkaloid, usaramine, which was reported in *Crotalaria* species (Figure 2) [13,14,15]. Being aware that retrosine (Figure 2) is frequently isolated from *Crotalaria* and differs from usaramine by the configuration of the double bond(*E* for usaramine and *Z* for retrosine), the ^1^H NMR data of compound **1**—and especially the chemical shift of the olefinic proton bearing the methyl group—were compared to those of usaramine and retrosine. The chemical shift of the indicated proton in compound **1** appears at 6.5 ppm, which is in full synchronization with the configuration of usaramine (~6.5 ppm in usaramine versus ~5.8 pp in retrosine). [13,16]. Compound **2** was analyzed and found to belong to pyrrolizidine-derived alkaloids and to be structurally close to compound **1** (MS and ^1^H NMR are provided in Supplementary Materials) [13]. The compound was assigned the molecular weight and molecular formula of 335 g/mol and C_18_H_25_NO_5_, respectively, as determined by HRMS.. Unfortunately, the full structure was not determined, due to the instability of the compound in solution at room temperature. Indeed, during the 2D experiments, which take longer than ^1^H NMR, the compound decomposes as evidenced by the appearance of signals that were not present in the ^1^H NMR spectrum. According to the molecular weight and the molecular formula, compound **2** could be senecionine and integerrimine, however, the ^1^H NMR profile did not match with either structures, based on the literature [13] (Figure 2).

## 3. Discussion

The use of plants in preparation of popular herbal drinks is frequent in many regions of the world. Beverages include infusion or decoction of herbals, spices, fruits, or other plant materials [17]. It is known that seeds of *Crotalaria cleomifolia* are used in Madagascar in remedies in folk medicine in different forms of preparations, such as infusions and decoctions. The most popular use is its consumption as a coffee beans regardless of the presence of potential toxic components. Due to the lack of studies regarding the chemical composition of the seeds from *Crotalaria cleomifolia* and the strong assumption regarding the presence of toxic pyrrolizidine-derived alkaloids, we undertook the present study to shed light on the alkaloidal composition. To ensure that the popular drink contains pyrrolizidine-derived alkaloids, we prepared drinks according to standard preparation (about 10 g of powdered seeds in 200 mL of boiled water for 10 min). A sample was lyophilized and analyzed by ^1^H NMR, showing the presence of typical signals for pyrrolizidine-derived alkaloids. The latter observation was confirmed following the specific extraction of alkaloids that allowed the isolation and characterization of usaramine (compound **1**) and its close analog (compound **2**).

Based on the quantities of pure compounds isolated, the seeds may contain over 0.16% of toxic compounds. The quantity of recovered pyrrolizidine-derived alkaloids in the drinks are dependent on different factors—the quantity of seeds used, the volume of water and its temperature, and the time of boiling. Moreover, it was evident that the observed yield of 0.16% was the most pessimistic, since certain compounds were not extracted and considerable loss may occur during the extraction and purification steps. According to the late regulatory requirement (May 2021) established by the Herbal Medicinal Products Committee, the tolerated amount of pyrrolizidine alkaloids in herbal medicinal plants/per day should not exceed 1.0 mg [18]. Based on this recommendation, the observed yield of 0.16% (160 mg/g) was extremely high and potentially highly toxic with regards to the quantities used to make daily beverages (at least few grams of seeds per beverage).

**Figure 3:**
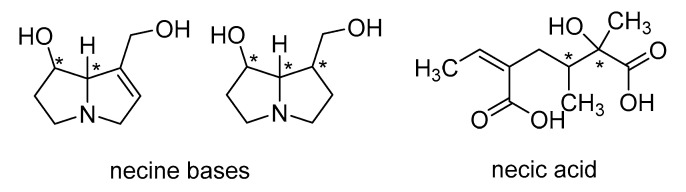
Scaffolds involved in the biosynthesis of macrocyclic pyrrolizidine-derived alkaloids. The stars (*) indicate that both enantiomers may exist. The carbon–carbon double bond of necic acid can be found in the *Z* and *E* configuration.

Pyrrolizidine-derived alkaloids are frequently isolated as macrocyclic dilactones, using a combination of a pyrrolizidine (necine base) with necic acid to produce macrocyclic rings with a range of sizes (Figure 3) [19]. In addition to macrocyclic dilactones, mono and diesters of necine as open chains, such as lycopsamine and echimidine, were reported [19].

Macrocyclic alkaloids derived from pyrrolizidine are produced by the plants as defense tools against herbivores. No therapeutic applications were linked to these compounds but rather their toxicity is often put in the spotlight. They are considered to be phytotoxins that present a high risk for humans and grazing livestock [5,6,20,21]. In mammals, they may cause severe hepatotoxicity [22,23], pneumotoxicity [24], and mutagenic effect [25,26]. The toxicity was mostly due to the bio-activation by cytochrome P450 (CYP) enzymes in liver, to achieve the hydrolysis of dilactones and release the necine bases [27,28].

It was reported that livestock fed with pyrrolizidine alkaloid-containing plants, undergo severe lung, liver, and kidney tissue damage [20]. One relevant case was recently reported from Brazilian farmers showing that horses fed with oats contaminated with *Crotalaria* seeds died due to severe liver failure [26]. The high level of hepatotoxicity and carcinogenic effect associated with this class of compounds presents a serious human health problem. Hence, any consumption of seeds of *Crotalaria cleomifolia* should be banned, as such use could be associated with a high degree of toxic effect.

## 4. Materials and Methods

### 4.1. Materials

Pods of *Crotalaria cleomifolia* were harvested in May 2014 at Manjakandriana, near Antananarivo (geographical coordinates, south: 18°54′06.9′′, East: 047°45′08.1′′). Seeds were separated from pods then dried at room temperature in the shadow. After drying, they were crushed in a fine powder using an electric grinder. Solvents used for the extraction were purchased from Cooper (Paris, France) and were used as received. Reagents used for the qualitative studies were purchased from Sigma-Aldrich (Saint Quentin Fallavier, France).

^1^H and ^13^C NMR (1D and 2D) spectra were measured on a Bruker Avance 400 MHz (Mannheim, Germany) NMR spectrometer in CDCl_3_ or CD_3_OD. Chemical shifts are reported in parts per million (δ) and coupling constants (*J*) in Hz. The residual CDCl_3_ or CD_3_OD were used as internal standards for ^1^H and ^13^C NMR, respectively. IR spectra were measured on a Perkin Elmer 1600 spectrophotometer. Optical rotations were measured at room temperature using a Perkin Elmer 343 Plus polarimeter. Electrospray ionization (ESI) mass spectra were recorded at the Institut de Chimie Moléculaire de Grenoble on a Waters Xevo G2-S Q TOF instrument (Guyancourt, France) with a nanospray inlet.

### 4.2. Methods

#### 4.2.1. Extraction of Alkaloids

Extraction in acidic media. A total of 30 g of the seeds powder were mixed with hydrochloric acid (1M, 30 mL), suspended in methanol (100 mL) and stirred for 24 h at room temperature. After filtration under vacuum, methanol was evaporated under reduced pressure at 30 °C. The aqueous solution was treated with ammonium hydroxide (20%) until pH 10. The solution was extracted with dichloromethane (3 × 20 mL). The organic phase was dried over sodium sulfate, filtered, and evaporated to dryness, to afford the crude extract (136 mg).

Extraction in basic media. A total of 20 g of the seeds powder were treated with 20 mL of ammonium hydroxide (20%) and mixed with dichloromethane (300 mL), then stirred at room temperature for 24 h. After filtration, the solution was evaporated under reduced pressure, the obtained residue was dissolved in 20 mL of distilled water, and sulfuric acid (10%) was added until reaching pH = 2. The solution was extracted with diethyl ether. The aqueous solution was treated with ammonium hydroxide (20%) until pH = 10. The basic solution was extracted with dichloromethane (3 × 20 mL). The organic solution was washed with water, dried over sodium sulfate, and evaporated to dryness to afford the crude residue (66 mg). A comparative TLC (silica gel, eluent: CH_2_Cl_2_/CH_3_OH 9:1) and revelation under UV light at 254 nm showed that the two extracts presented the same profile. Hence, the two extracts were mixed.

#### 4.2.2. Determination of the Presence of Alkaloids in the Extracts

The two extracts highlighted above were subjected to qualitative chemical screening for identification of alkaloids. To this end, we used Wagner, Dragendorff, and Mayer tests [26]. Both extracts tested were positive, indicating the presence of alkaloids in crude extracts.

#### 4.2.3. Purification of the Extract

The crude extract (200 mg) was purified on a silica gel chromatography column, using a gradient of dichloromethane:methanol: 98:2; 95:5; 90:10, and 0:100. In total, 35 fractions (10 mL each) were collected, analyzed by TLC using dichloromethane/methanol—92/8 as eluent then concentrated. Fractions 5 (44 mg) and 8 (39 mg) showed the presence of two compounds in pure forms, here compounds **1** and **2**.

Spectral characterization of compound **1**. ${\left[C\right]}_{D}^{25}\;$+6.7° (c 0.009, MeOH); IR (CHCl_3_) 3466, 3059, 2971, 2845, 1716, 1443, 1273, 1213, 1158 cm^−1^; ^1^H NMR (CD_3_OD): δ 6.50 (q, *J* = 7.2 Hz, 1H), 6.22 (s, 1H), 5.70 (d, *J* = 11.5 Hz, 1H), 5.15 (bs, 1H), 4.30 (bs,1H), 4.21 (d, *J* = 11.4 Hz, 1H), 3.96 (d, *J* = 16 Hz, 1H), 3.61 (m, 2H), 3.40 (dd, *J* = 5, 15.2 Hz, 1H), 3.25 (t, *J* = 8 Hz, 1H), 2.61 (m, 2H), 2.48–2.0 (m, 3H), 1.70 (m, 1H), 1.80 (m, 1H), 1.83 (m, 1H), 1.74 (d, *J* = 7 Hz, 3H), 0.82 (d, *J* = 7.1 Hz, 3H). ^13^C NMR (CD_3_OD) δ 176.1, 170.5, 136.6, 136.5, 135.1, 133.6, 82.5, 77.9, 76.9, 68.2, 63.4, 61.2, 53.9, 38.1, 34.4, 30.4, 14.3, 12.4; MS: HRMS calcd. for C_18_H_26_NO_6_ 352.1660 (M + 1), obsd. 352.1755.

## 5. Conclusions

Specific extractions were conducted on the seeds of *Crotalaria cleomifolia* and revealed the presence of two pyrrolizidine-derived alkaloids, from which one was identified as usaramine. The latter is known for its high hepatotoxicity, as evidenced by in vivo studies. Hence, any consumption of the seeds of *Crotalaria cleomifolia* should be banned, as such use could be associated with a high degree of intoxication.

## Data Availability

Not applicable.

## Supplementary Materials

The following are available online. Figure S1. MS, 1D, and 2D NMR data of compound **1** and ^1^H NMR of compound **2**.

## Sample Availability

Samples of the seeds of *Crotalaria cleomifolia* are availbale from authors.
