# Supplementary material for: Pyrrolizidine-Derived Alkaloids: Highly Toxic Components in the Seeds of Crotalaria cleomifolia Used in Popular Beverages in Madagascar
Source: Molecules. 2021 Jun 7;26(11):3464. doi: 10.3390/molecules26113464 (PMC8201287; doi:10.3390/molecules26113464)

Supplementary Material

Pyrrolizidine-derived Alkaloids: Highly Toxic Components in the Seeds of *Crotalaria cleomifolia* Used in Popular Beverages in Madagascar

IR, MS and NMR data (^{1}H NMR, ^{13}C NMR, COSY, HMBC and HMQC) of compound **1**.

MS and ^{1}H NMR of compound **2**.

IR of compound **1**.

Results of Peak Find

No.	Position	Intensity
1	3466.42	38.0251
2	3349.75	40.9304
3	3059.51	39.3021
4	2971.77	32.099
5	2933.2	31.4473
6	2878.24	35.5924
7	2845.45	32.3786
8	2696	44.4849
9	2360.44	50.2095
10	2341.16	51.7481
11	1716.34	15.843
12	1654.62	34.4802
13	1610.27	46.7193
14	1443.46	35.7835
15	1382.71	37.6886
16	1362.46	43.1476
17	1346.07	43.6109
18	1323.89	47.4271
19	1273.75	16.5852
20	1213.01	17.2559
21	1158.04	16.221
22	1123.33	26.0808
23	1105.01	32.784
24	1080.91	32.0401
25	1059.69	35.6271
26	1021.12	32.4405
27	999.91	32.9675
28	981.59	45.5112
29	970.019	47.5729
30	948.806	41.5148
31	908.308	44.2201
32	863.953	50.3352
33	827.312	44.2218
34	814.777	47.704
35	778.136	44.8127
36	752.102	41.9905
37	732.817	27.8814
38	715.461	41.8062
39	700.998	35.7793
40	665.321	30.2707
41	645.072	34.5808
42	596.861	18.6228

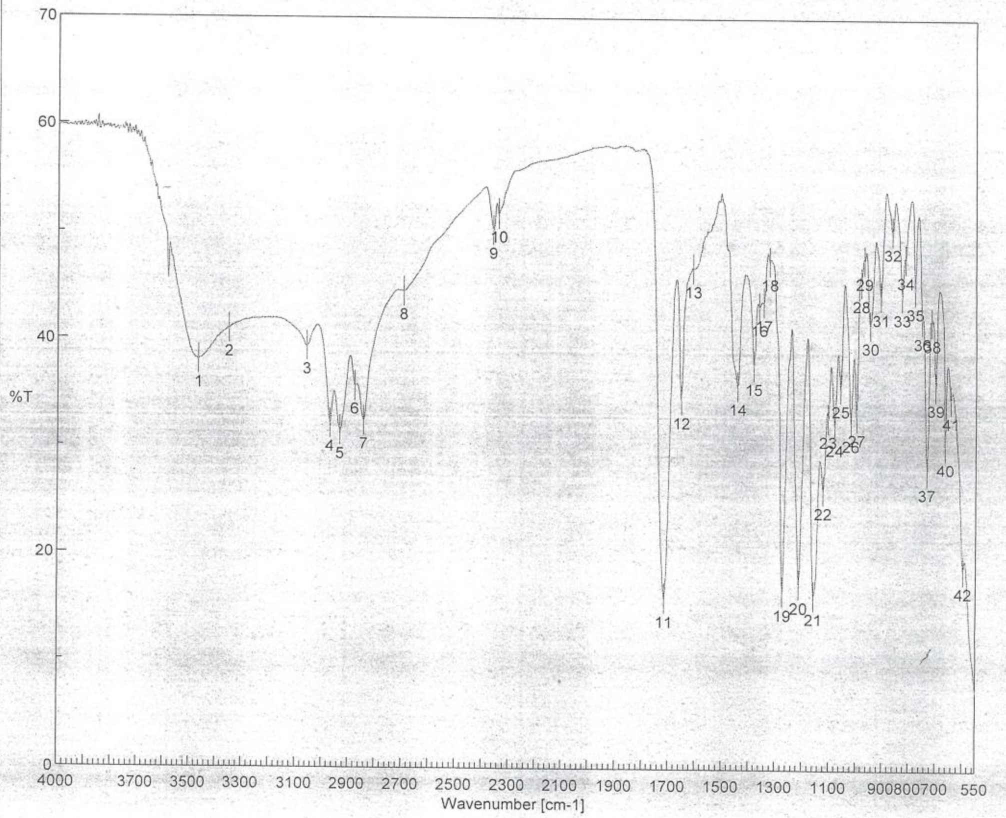

MS of compound **1**.

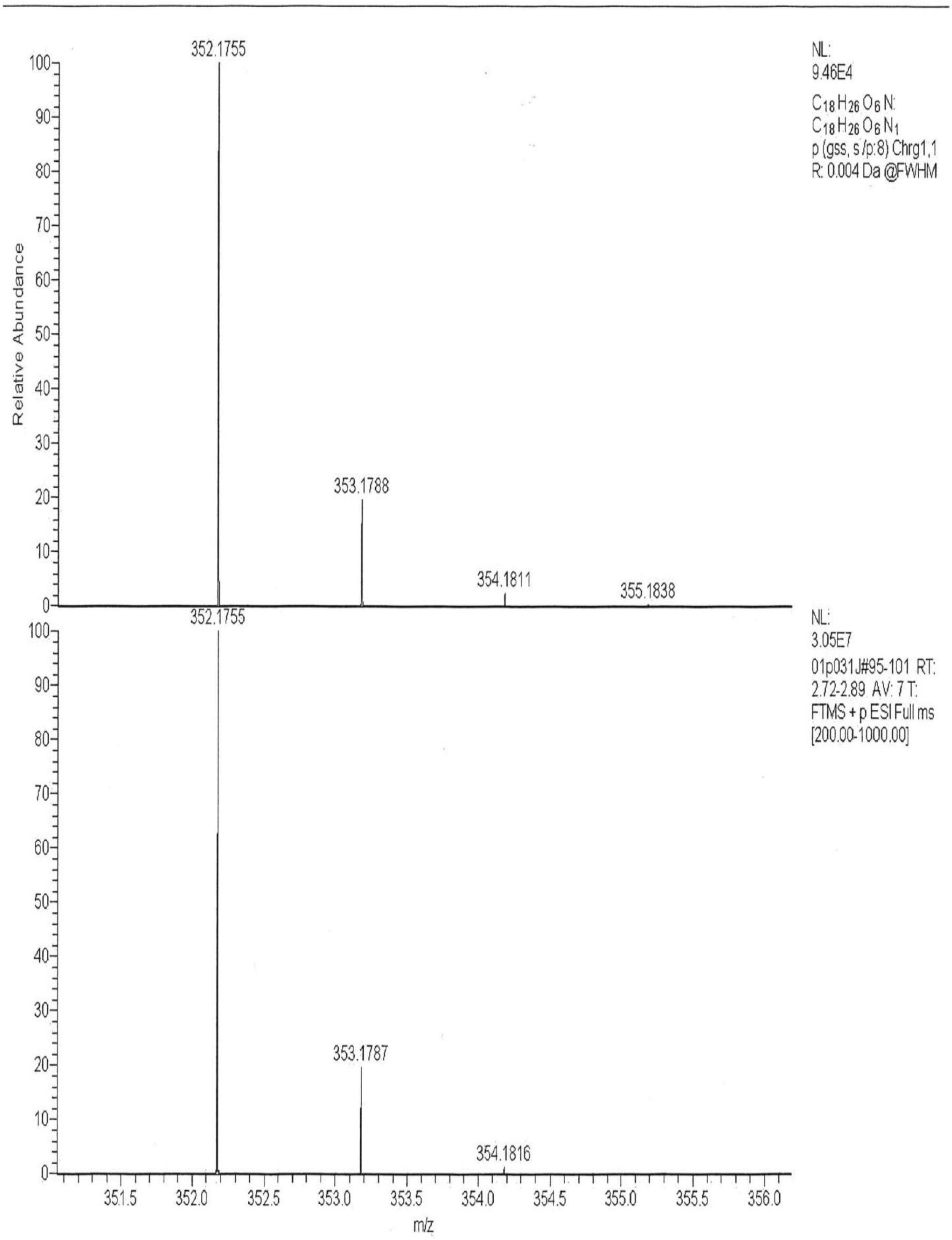

^{1}H NMR of compound **1**.

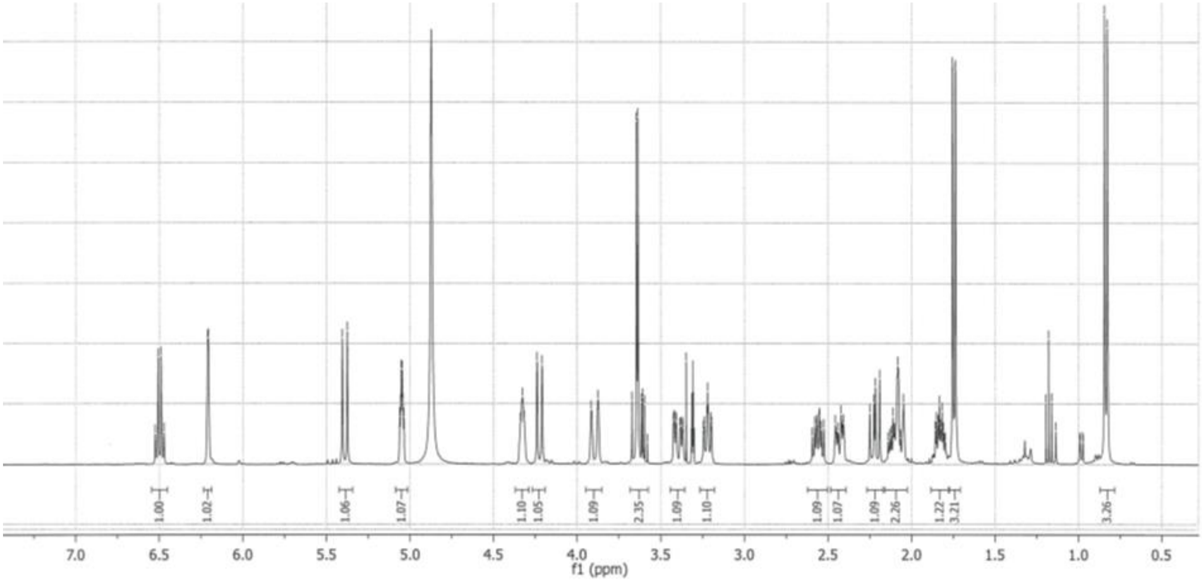

^{13}C NMR of compound **1**.

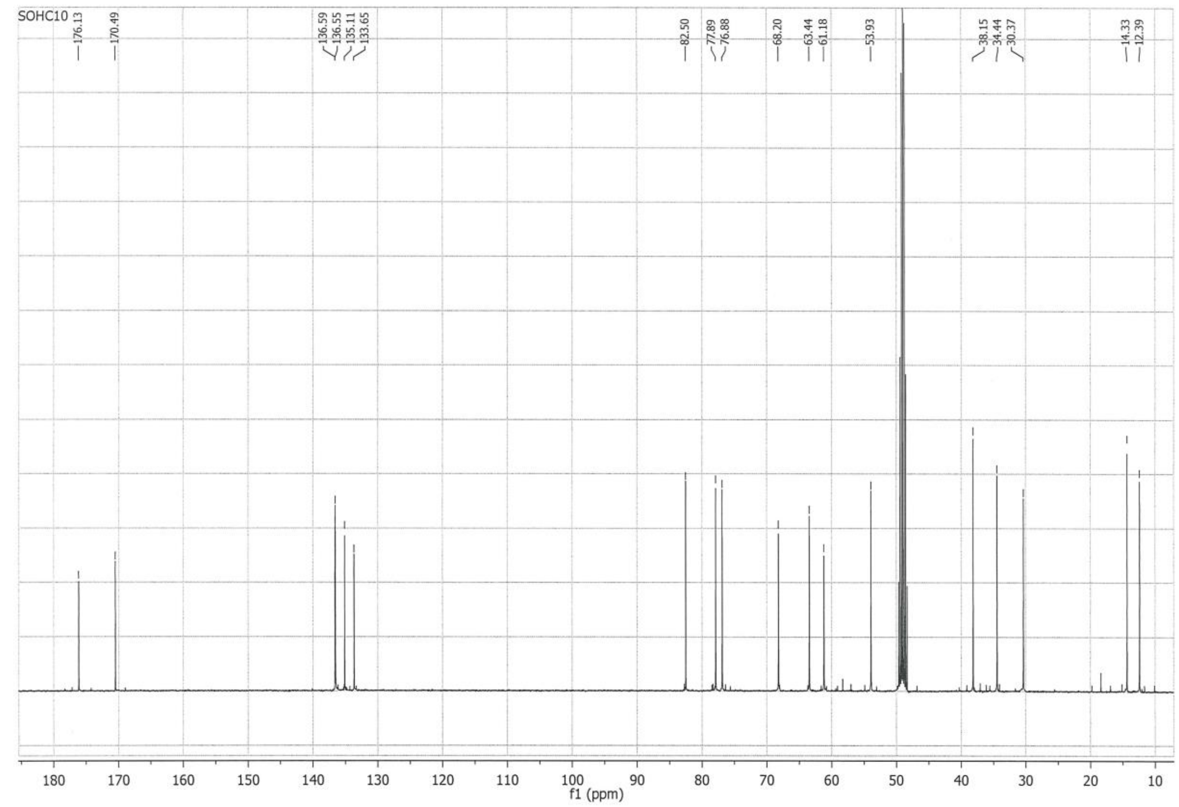

COSY of compound **1**.

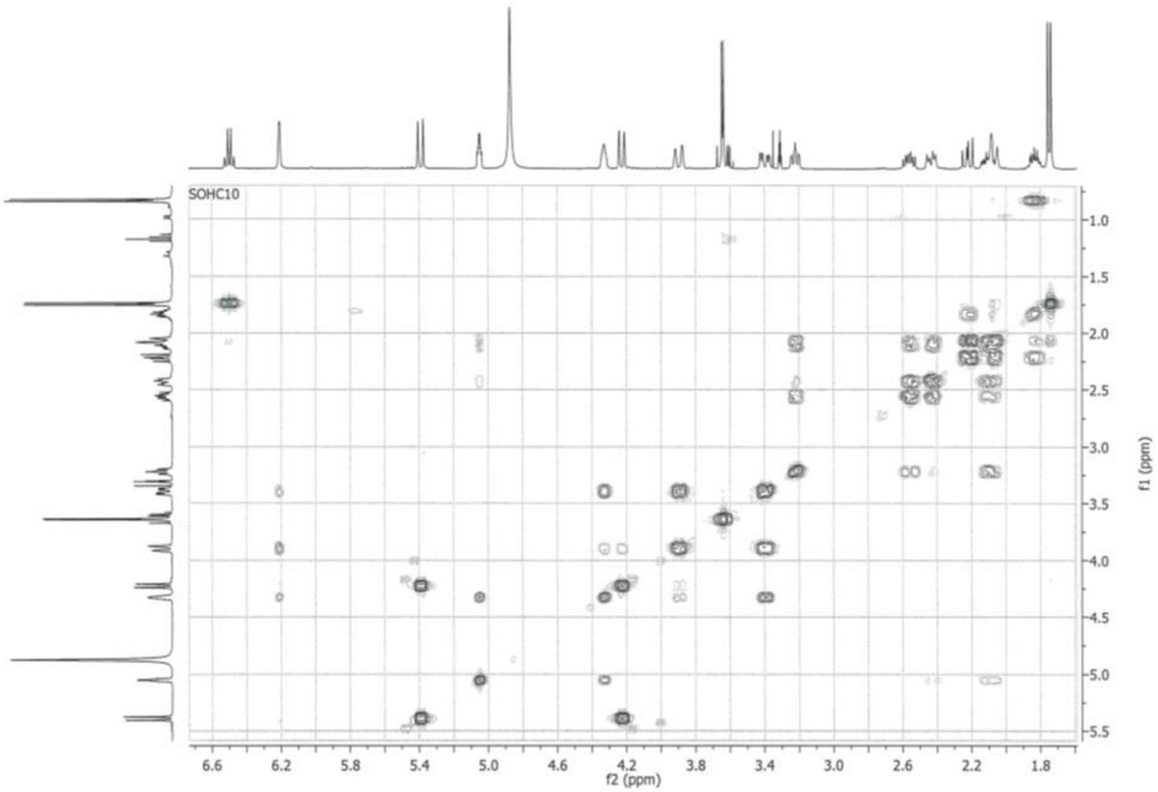

HMBC of compound **1**.

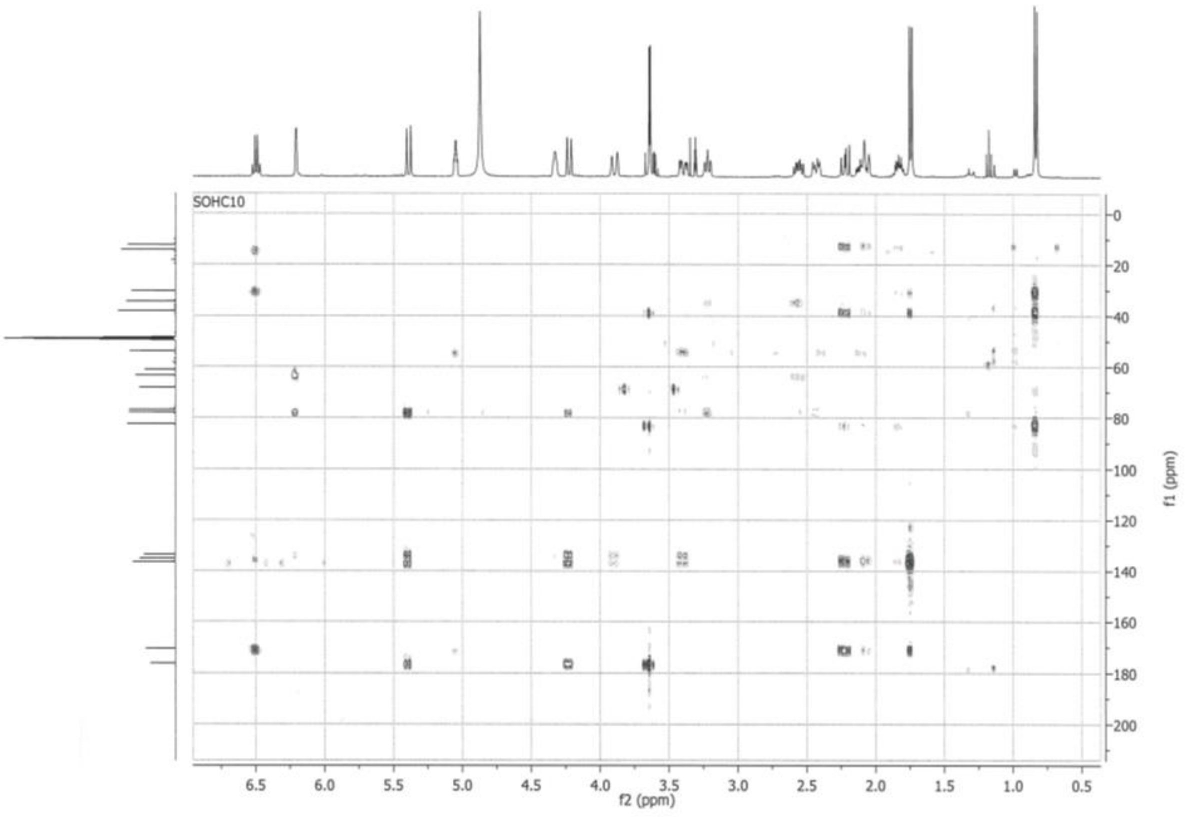

HMQC of compound **1**.

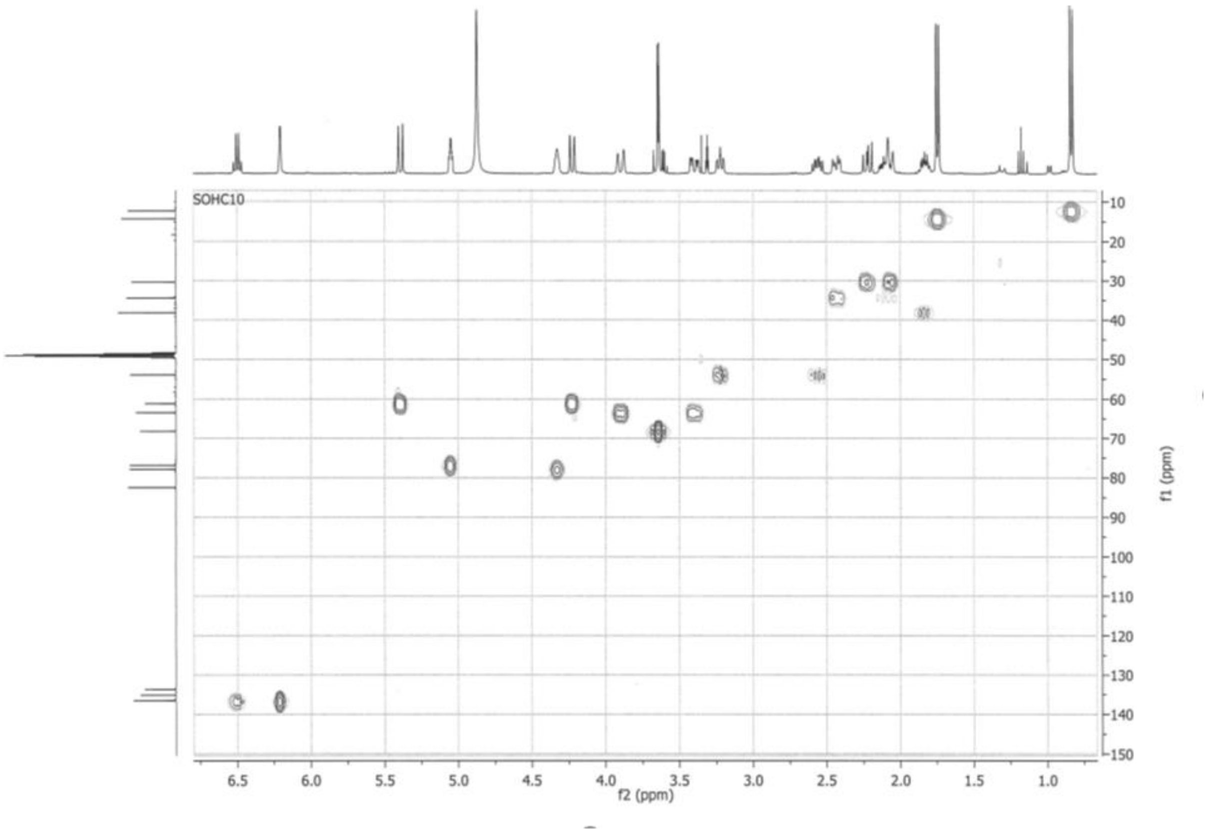

MS of compound **2**.

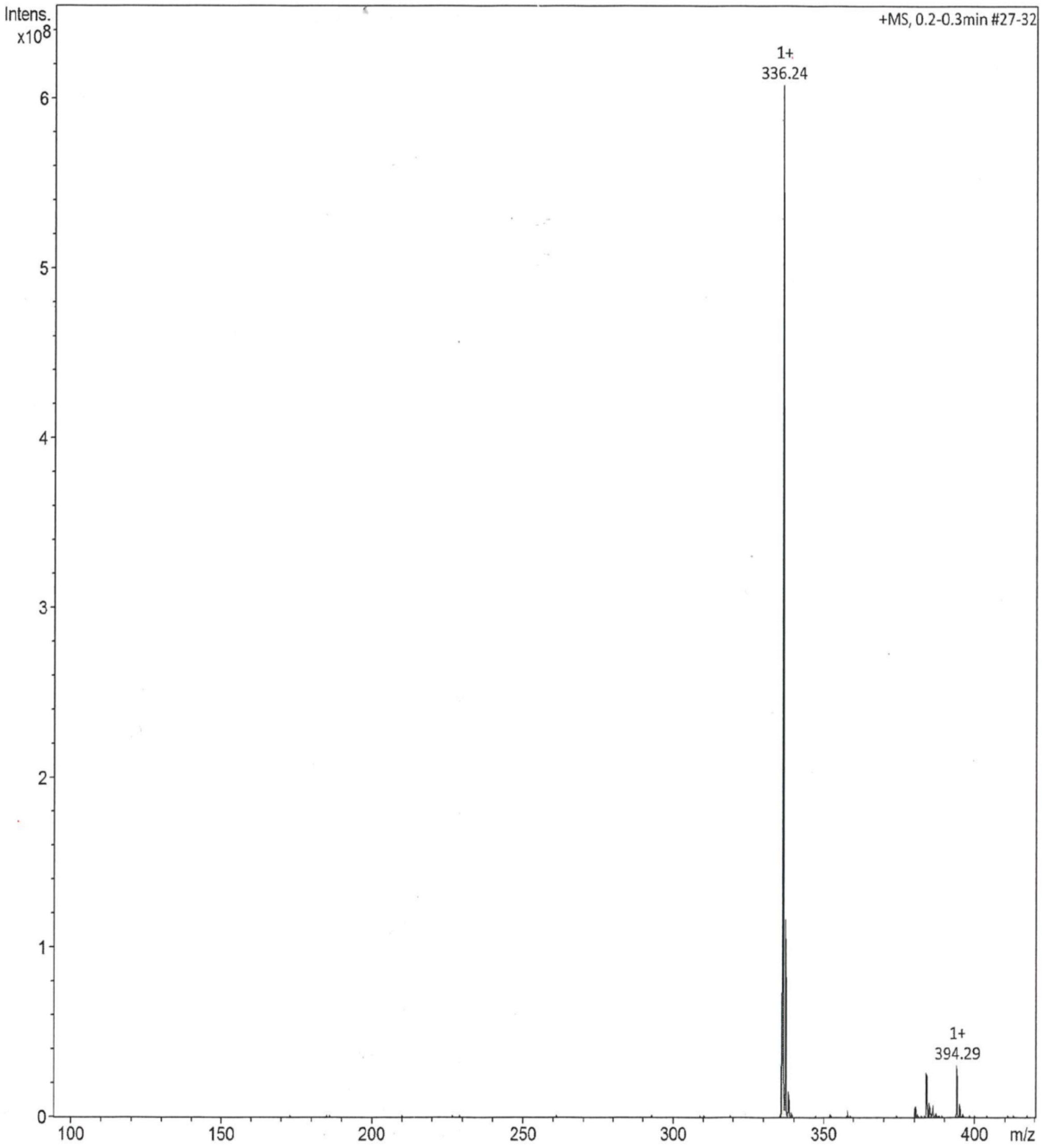

^1H NMR of compound **2**.

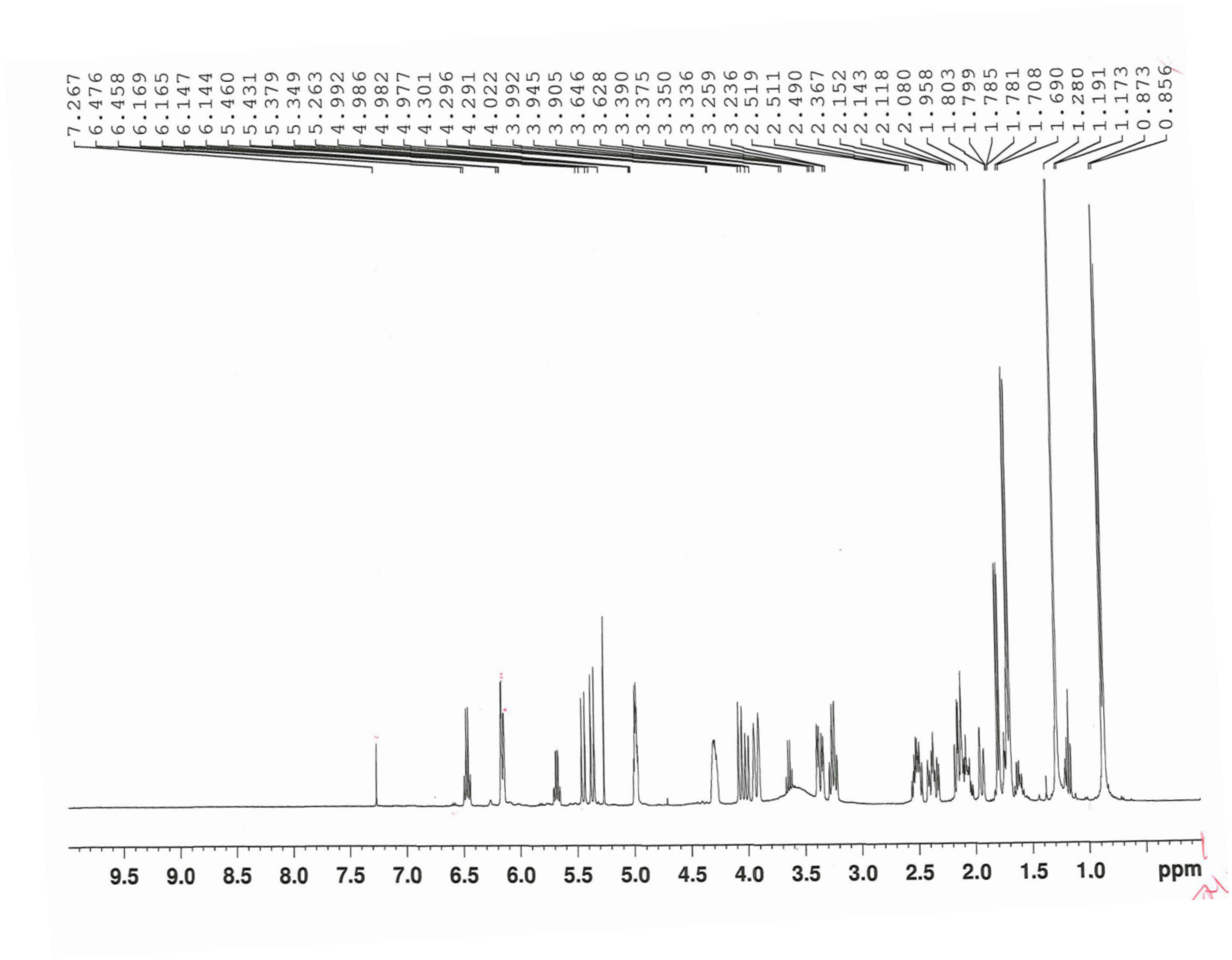

Supplement: Supplementary file 1 [file molecules-26-03464-s001.zip › molecules-1246564-supplementary.pdf]
